# *Zygosaccharomyces rouxii*, an Aromatic Yeast Isolated From Chili Sauce, Is Able to Biosynthesize 2-Phenylethanol *via* the Shikimate or Ehrlich Pathways

**DOI:** 10.3389/fmicb.2020.597454

**Published:** 2020-10-29

**Authors:** Jun Dai, Ke Li, Na Song, Wanting Yao, Huili Xia, Qiao Yang, Xiaoling Zhang, Xin Li, Zhi Wang, Lan Yao, Shihui Yang, Xiong Chen

**Affiliations:** ^1^Key Laboratory of Fermentation Engineering (Ministry of Education), National “111” Center for Cellular Regulation and Molecular Pharmaceutics, Hubei Provincial Cooperative Innovation Center of Industrial Fermentation, College of Bioengineering, Hubei University of Technology, Wuhan, China; ^2^ABI Group, College of Marine Science and Technology, Zhejiang Ocean University, Zhoushan, China; ^3^State Key Laboratory of Biocatalysis and Enzyme Engineering, School of Life Sciences, Hubei University, Wuhan, China

**Keywords:** aroma-producing strain, *Zygosaccharomyces rouxii*, 2-phenylethanol, Shikimate pathway, Ehrlich pathway

## Abstract

We isolated an aromatic strain of yeast (M2013310) from chili sauce. Assembly, annotation, and phylogenetic analysis based on genome sequencing, identified M2013310 as an allodiploid yeast that was closely related to *Zygosaccharomyces rouxii*. During fermentation, M2013310, produced an aromatic alcohol with a rose-honey scent; gas chromatography tandem mass spectrometry identified this alcohol as 2-phenylethanol. The concentration of 2-phenylethanol reached 3.8 mg/L, 1.79 g/L, and 3.58 g/L, in M3 (NH_4_^+^), M3 (NH_4_^+^ + Phe), and M3 (Phe) culture media, after 72 h of fermentation, respectively. The mRNA expression levels of *ARO8* encoding aromatic aminotransferases I and *ARO10* encoding phenylpyruvate decarboxylase by M2013310 in M3 (Phe) were the lowest of the three different forms of media tested. These results indicated that M2013310 can synthesize 2-phenylethanol via the Shikimate or Ehrlich pathways and the production of 2-phenylethanol may be significantly improved by the over-expression of these two genes. Our research identified a promising strain of yeast (M2013310) that could be used to improve the production of 2-phenylethanol.

## Introduction

The *Zygosaccharomyces* genus consists of six different species: *Z. bailii*, *Z. bisporus*, *Z. kombuchaensis*, *Z. lentus*, *Z. mellis*, and *Z. rouxii* (Zuehlke et al., 2013). *Z. rouxii* is a halotolerant and osmotolerant species of yeast that is most phylogenetically related to *Saccharomyces cerevisiae* (Kobayashi and Hayashi, 1998; Solieri et al., 2013; Guo et al., 2020). It is known that *Z. rouxii* has different genomic forms, including haploid and allodiploid forms, at least (Kinclová et al., 2001; Solieri et al., 2013; Watanabe et al., 2017). *Z. rouxii* CBS732 is a haploid strain featuring one copy of each gene (Montigny et al., 2000; Kinclová et al., 2001). ATCC 42981, isolated from miso paste, features a mosaic genome with two copies of many genes and represents a sterile allodiploid (Kinclová et al., 2001; Bizzarri et al., 2016). Generally, the *Z. rouxii* strain of yeast is applied during the fermentation process used to make soy sauce and miso paste (Kobayashi and Hayashi, 1998; Dakal et al., 2014) and can produce a range of different volatile compounds, including ethanol, ethyl propanoate, 1-butanol, ethyl 2-methylpropanoate, 4-hydroxy-2-ethyl-5-methyl-3(2H)-furanone (HEMF), and 2-phenylethanol (Lee et al., 2014).

2-Phenylethanol (2-PE) is a higher aromatic alcohol that is characterized by its rose-honey-like fragrance and has been utilized as a fragrance ingredient in a range of different products, including cosmetics, perfumes, beer, olive oil, tea, and coffee (Scognamiglio et al., 2012; Chreptowicz et al., 2016). Furthermore, 2-PE plays an important role in the pharmaceutical industry because it can exert antibacterial effects on Gram-negative bacteria, coccus, bacillus, and some fungi (Fraud, 2003). Natural forms of 2-PE are extracted from aromatic essential plant oils, including rose, jasmine, or hyacinth; however, it is difficult to satisfy market demand and the commercial price of this extraction process is high (approximately $1,000/kg). Over recent years, the majority of global 2-PE production involved a chemical process that is far less extensive ($5/kg). However, this chemical process is limited by the fact that it involves benzene and styrene (known carcinogens) and produces byproducts that are difficult to remove (Etschmann et al., 2002; Hua and Xu, 2011). Therefore, 2-PE is now synthesized mostly by microbial fermentation; this is far more cost-effective and provides much simpler and more efficient options for product purification. Bacteria such as *Enterobacter* sp. CGMCC 5087 (Zhang et al., 2014), and fungi such as *Aspergillus oryzae* (Masuo et al., 2015), are able to successfully produce 2-PE, but with relatively low yield.

The microorganisms that are most efficient at producing 2-PE are yeasts, including *S. cerevisiae, Kluyveromyces marxianus*, *Kluyveromyces lactis, Pichia fermentans*, *Candida glycerinogenes*, and *Z. rouxii* (Etschmann et al., 2003; Kim et al., 2014; Lu et al., 2016; Chreptowicz et al., 2018; Martínez-Avila et al., 2019). Yeasts are known to predominantly biosynthesize 2-PE *via* the Shikimate or Ehrlich pathways (Figure 4) (Wang et al., 2019). The Shikimate pathway is a long pathway with multiple branches and a variety of inhibitory feedback mechanisms (Etschmann et al., 2002); this pathway is associated with low yields of 2-PE. However, when using amino acids as the sole source of nitrogen, the Ehrlich pathway is far more efficient; consisting of three steps, this pathway is very effective in synthesizing 2-PE.

In the present study, we isolated an aromatic strain of yeast (M2013310) from chili sauce. This yeast produced a rose-honey-like fragrance during fermentation. The strain was identified as *Z. rouxii* and was able to synthesize 2-PE. Next, we used L-Phe or ammonium sulfate as nitrogen sources to help us to investigate the pathways responsible for the biosynthesis of 2-PE in this particular strain.

## Materials and Methods

### Isolation of Yeast Strains and Culture Conditions

Strain M2013310 was isolated from chili sauce in our laboratory. First, the sauce sample was serially diluted with a sterile 0.85% (w/v) NaCl solution. These dilutions were then screened on yeast extract-peptone-glucose (YEPD; 10 g/L of yeast extract, 20 g/L of peptone, and 20 g/L of glucose) agar plates prepared with 15 g/L of agar and 180 g/L of NaCl. After incubation for 7 days at 30°C, individual colonies were isolated and purified by repeated streaking. Isolates were maintained on YEPD agar slants and kept at 4°C before preservation by freeze-drying. Yeast isolates were routinely sub-cultured in YEPD at 30°C for 72 h with shaking at 200 rpm.

Strain M2013310 was cultivated in 50 mL of YEPD medium and activated in 250 mL flasks at 30°C with shaking at 200 rpm. Subsequently, 2.5 mL of secondary activated cells grown to mid-log phase and inoculated into M3 (Phe) based on Mierzejewska et al. (2017), M3 (NH_4_^+^), and M3 (NH_4_^+^ + Phe) culture media (Table 1), respectively. These were incubated at 30°C with shaking at 200 rpm for 6 h, 12 h, 24 h, 36 h, 48 h, or 72 h (in triplicate).

**TABLE 1:** The compositions of the cell culture media used to produce 2-PE in this study.

	Glucose (g/L)	Sucrose (g/L)	YNB (g/L)	I-Phe (g/L)	(NH_4_)_2_SO_4_ (g/L)	MgSO_4_7H_2_O (g/L)
M3 (Phe)	30	8	1.7	9	–	0.5
M3 (Phe + NH_4_^+^)	30	8	1.7	4.5	2.25	0.5
M3 (NH_4_^+^)	30	8	1.7	–	4.5	0.5

*Yeast nitrogen base (YNB) without amino acids and ammonium sulfate (BD Co., Ltd.).*

### Morphological and Physiological Analysis

Isolates were characterized using established criteria for spore formation and the physiological tests described by Kurtzman et al. (2011). Cell morphology was examined by optical microscopy. Sugar fermentation and assimilation tests were also performed using the VITEK system with YST cards, in accordance with the manufacturer’s instructions (bioMérieux). The effects of various culture media on cell growth were examined in test tubes containing 10 ml of liquid medium; these were inoculated with approximately 10^5^ cells/mL. Tubes were then incubated under both static and shaking conditions (200 rpm) for 7 days. The ability of the isolates to grow at different temperatures (4, 8, 16, 20, 28, 34, 37, and 40°C) was then evaluated using YEPD medium. In order to test the effects of high sugar concentrations on growth, we supplemented the YEPD medium with different amounts of glucose (200 g/L, 400 g/L, and 600 g/L) and incubated these cultures at 28°C. We also investigated growth in modified YEPD (mYEPD), which lacked glucose but contained fructose (20 g/L), at 28°C. Cell density was monitored by measuring OD_600_.

### Genome Sequencing, Assembly, and Annotation

Genomic DNA was extracted from strain M2013310 from pure cultures and sequenced on a PacBio single-molecule real-time (SMRT) Sequel sequencer. *De novo* genome assembly of the PacBio reads was then carried out using the hierarchical genome-assembly process (HGAP4) with default parameters, including consensus polishing with Quiver (Chin et al., 2013). Augustus (version 3.3) was used for gene prediction (Stanke and Morgenstern, 2005), and non-coding RNA was identified by sequence alignment with the Rfam database (version 12.0) (Gardner et al., 2009). Gene functional annotation was performed by aligning the protein sequences to the National Center for Biotechnology Information Non-redundant protein sequences (NCBI NR), Clusters of orthologous groups for eukaryotic complete genomes (KOG), and Kyoto Encyclopedia of Genes and Genomes (KEGG) databases, using BLASTP v2.3.0+ with an E-value cut-off of 1 × 10^–5^.

### Phylogenetic Analysis

Orthologous and paralogous gene families were assigned from six species (*Z. rouxii*, *Z. bailii*, *Z. parabailii*, *S. cerevisiae*, *S. eubayanus*, and *S. arboricola*) by OrthoFinder (Emms and Kelly, 2015) with default parameters. Gene families that contained only one gene for each species were selected to construct a phylogenetic tree. The protein sequences of each gene family were independently aligned by Muscle v3.8.3 (Edgar, 2004) and then concatenated into one super-sequence. The phylogenetic tree was constructed by maximum likelihood (ML) using PhyML v3.0 (Guindon et al., 2010; Darriba et al., 2011).

### Determination of Volatile Flavor Components

Gas chromatography tandem mass spectrometry (1200 L GC/MS-MS; Varian Company, United States) was used to detect volatile flavor components. Chromatography included a DB-WAX (30 m × 0.25 mm × 0.25 μm) capillary column. Helium was used as a carrier gas (flow rate: 0.8 mL/min). The initial temperature was 40°C; this was maintained for 4 min. A 6°C/min rate was then used to reach 160°C, and a 10°C/min rate to reach 220°C; this was maintained for 6 min. Mass spectrometry involved an interface temperature of 250°C, an ion source temperature of 200°C, the EI ionization mode, an electron energy of 70 eV, a detection voltage of 350 V, and an emission current of 200 μA.

### The Growth of *Z. rouxii* M2013310 in the Presence of Exogenous 2-PE

In brief, 2.5 mL of the secondary activated strain of *Z. rouxii* M2013310 was inoculated into 50 mL of fresh YEPD medium in five 250 mL flasks and cultivated at 30°C with shaking at 200 rpm. When the cultures achieved an OD_600_ of 0.8, we added 2-PE to five of the flasks to a final concentration of 1, 2, 3, 4, 5 g/L. The sixth flask acted as a control and did not contain 2-PE. Cultures were incubated for 24 h, 48 h, or 72 h (in triplicate) and growth was monitored by the measurement of OD_600_ measurement.

### Growth, Glucose Assays, L-Phe, and 2-PE Fermentation Analysis

In brief, 5 ml of each culture was centrifuged at 8000 rpm for 3 min at 4°C. We then discarded the supernatant and added an equivalent volume of deionized water. The OD_600_ was then determined using a spectrophotometer. Glucose consumption was determined by the DNS method (Deed et al., 2018). One milliliter of culture was centrifuged for 10 min at 12000 rpm at 4°C. The remaining supernatant was then used to determine the concentration of 2-PE and L-Phe, which were both quantified by high performance liquid chromatography (Thermo Fisher Scientific) with a C-18 column. A solvent, consisting of ultra-pure water/methanol (40/60), or ultra-pure water/methanol (50/50), was applied for the analysis of 2-PE or L-Phe, respectively, with a constant flow rate of 0.6 mL/min or 1 mL/min. We then estimated the concentrations of 2-PE and L-Phe at 210 nm and 260 nm, respectively.

### Quantitative Real-Time PCR (qRT-PCR)

Total RNA was isolated from yeast cells with a total RNA extraction kit (Tiangen Biochemical Technology Co., Ltd.). We then used qRT-PCR to determine the relative mRNA expression levels of *GAP1*, *ARO8*, *ARO9*, *ARO10*, or *ENO1*. The reaction mixture for reverse transcription included 1μg of total RNA, 4 μL of 4 × gDNA wiper Mix, 4 μL of 5 × HiscriptIIqRT SuperMix II, and RNase free ddH_2_O (Vazyme Biotech Co., Ltd.). PCR was performed at 50°C for 15 min and 85°C for 5 s. A 20 μL reaction mixture was prepared for each qPCR reaction and contained 10 μL of ChamQ Universal SYBR qPCR Master Mix, gene-specific primers (Table 2), 1 μL of Temple DNA/cDNA, and RNase free ddH_2_O. PCR was then performed at 95°C for 30 s, with 40 cycles of 95°C for 10 s and 60°C for 30 s, 95°C for 15 s, 60°C for 1 min, and 95°C for 15 s, using a QuantStudio 3 real-time PCR system (Thermo Fisher Scientific). Delta cycle threshold (ΔC_*T*_) values were calculated by the C_*T*_s of the target genes minus the C_*T*_ of *ENO1*, which was used as a housekeeping gene. ΔΔC_*T*_ values were calculated by ΔC_*T*_ values from the experimental samples – the C_*T*_ of the control sample. Fold changes were calculated using the 2^–Δ^ ^Δ^ ^CT^ method (Livak and Schmittgen, 2001).

**TABLE 2:** The primers used in this study.

Primers	Sequences
ENO F	5′-CGGTATGGACTGTGCTTCTTCTG-3′
ENO R	5′-GGATGGGTCGCTGTTAGGGTTCTT-3′
ARO9 F	5′-GGTATGCCCAATGCTGGCTTC-3′
ARO9 R	5′-CACTAGCGGCCTCATACCCTCAGTG-3′
ARO10 F	5′-TTACGCTGCTGATGGTTATTCTCGC-3′
ARO10 R	5′-CGGCAACACCATTTATCGCAC-3′
GAP1 F	5′-AAAGATTGCTATTGCTACCGCCAG-3′
GAP1 R	5′-AACGCAGTACCAGACCCAAC-3′
ARO8 F	5′-GCTCAAGGTGTTACTACCATTCC-3′
ARO8 R	5′-GACGTACCAGTTGGGTTTTGAC-3′

## Results and Discussion

### Phenotypic Characteristics

After 3 days of growth at 28°C in YEPD broth, cells from strain M2013310 were observed to adopt an ovoid or slightly elongated shape. These cells were 2.6–2.7 × 4.1–5.0 μm in diameter, non-flagellated, non-gliding, and appeared in pairs or in small groups. Colonies on YEPD agar were white in color, opaque, and circular, with regular margins after incubation for 3 days at 30°C. Growth occurred with 0–24% NaCl (w/v) at 6–34°C and at a pH of 4.0–7.0. Sugar fermentation and assimilation tests showed that the cultures were positive for leucine-arylamidase activity, D-glucose assimilation, D-mannose assimilation, and xylitol assimilation. However, the cultures were negative for L-lysine-arylamidase, tyrosine arylamidase, β-*N*-acetyl-glucosaminidase, γ-glutamyl-transferase, PNP-*N*-acetyl-beta-D-galactosaminidase, urease, α-glucosidase, esculin hydrolyze, L-malate assimilation, erythritol assimilation, glycerol assimilation, arbutine assimilation, amygdalin assimilation, D-galactose assimilation, gentiobiose assimilation, lactose assimilation, methyl-A-D-glucopyranoside assimilation, D-cellobiose assimilation, D-maltose assimilation, D-raffinose assimilation, D-melibiose assimilation, D-melezitose assimilation, L-sorbose assimilation, L-rhamnose assimilation, D-sorbitol assimilation, sucrose assimilation, D-turanose assimilation, D-trehalose assimilation, nitrate assimilation, L-arabinose assimilation, D-galacturonate assimilation, L-glutamate assimilation, D-xylose assimilation, DL-lactate assimilation, acetate assimilation, citrate (sodium) assimilation, glucuronate assimilation, L-proline assimilation, 2-keto-D-gluconate assimilation, *N*-acetyl-glucosamine assimilation, and D-gluconate assimilation. Morphological and physiological results demonstrated that strain M2013310 was characteristic of the species *Z. rouxii* (James, 2011).

### High-Quality Genome Assembly and Gene Annotation

We generated 7.3 gigabase (Gb) PacBio single-molecule real-time (SMRT) sequences with a mean read length of 3.9 kb. These PacBio SMRT sequences was assembled into 38 contigs with a total length of 18.6 Mb, an N50 length of 1.4 Mb, and an N90 length of 0.8 Mb (Table 3), via the hierarchical genome-assembly process (HGAP4).

**TABLE 3:** The assembly of the genome for strain M2013310.

Assembly feature	CCTCC M2013310
Assembled sequence (bp)	18,600,657
No. of scaffolds	38
Sequence depth	303.70
Maximum contig length (bp)	1,922,742
N50 length (bp)	1,437,955
N90 length (bp)	819,506
GC content in Genome (%)	39.9

**FIGURE 1:**
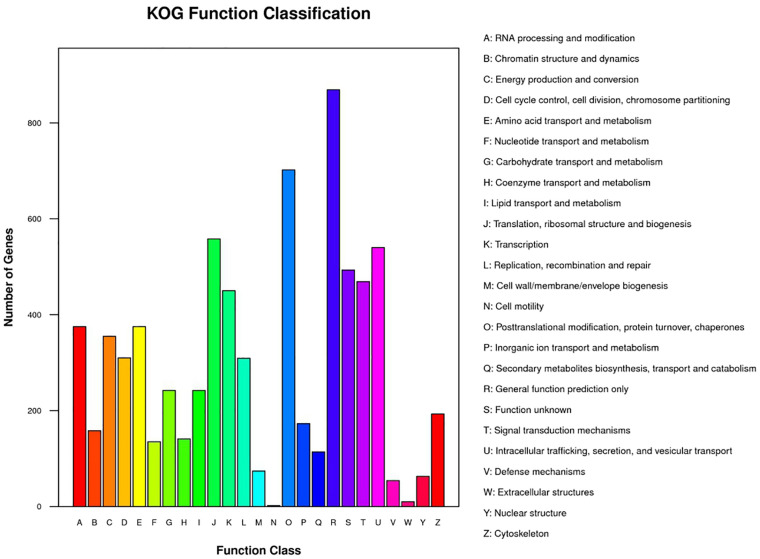
KOG function categories for strain M2013310.

The GC content in the genome of strain M2013310 was similar to that of *S. cerevisiae* S288c and *Z. rouxii* CBS732 (Table 4). Furthermore, the gene density and mean GC content in the sequence coding for amino acids in protein (CDS) of the M2013310 genome are similar with those described for other hemiascomycetous yeasts, including *S. cerevisiae* S288c and *Z. rouxii* CBS732 (Table 4). In total, 9,043 genes were predicted to be present in the genome of M2013310. This is approximately twice that of the genes annotated for *Z. rouxii* CBS732 (4,991 genes), 89.5% of these genes were considered to be duplicated genes (8,097 genes) as the proteins encoded share >70% identity and >70% coverage at the amino acid level (Supplementary Table S1). The total genome size of strain M2013310 (18.6 Mb) was well above the size expected for a haploid genome (type strain *Z. rouxii* CBS732, 9.7 Mb). These results indicated that the genome of M2013310 could be diploid. The 9,043 genes identified in strain M2013310 were functionally annotated using KOG function categories (Tatusov et al., 1997) (Figure 1 and Supplementary Table S2). Analysis showed that the highest number of genes were assigned to the functional categories of ‘general function prediction only’ (869 genes), ‘posttranslational modification, protein turnover, chaperones’ (702 genes), and ‘translation, ribosomal structure and biogenesis’ (558 genes). In addition, 493 genes were assigned to unknown functions. The vast majority of the proteins in strain M2013310 exhibited homologs with proteins found in yeast species which are phylogenetically close to species of *Z. rouxii*, including *Z. bailii*, *Torulaspora delbrueckii*, *S. cerevisiae*, and other yeasts of the *Saccharomycetaceae* family. These results showed that M2013310 may be an allodiploid yeast.

**TABLE 4:** The general features of strain M2013310, *Z. rouxii* CBS732, and *S. cerevisiae* S288c genomes.

Strain	CCTCC M2013310	*Z. rouxii* CBS732	*S. cerevisiae* S288c
Ploidy	(∼2n)	n	n
Genome size (Mb)	18.6	9.7	12.3
GC content in genome (%)	39.9	39.1	38.3
Total number of CDS	9,043	4,991	5,769
GC content in CDS (%)	40.7	40.2	40.3
Average CDS length (bp)	1,507	1,491	1,464

### Phylogenic Analysis Based on Genome Sequences

**FIGURE 2:**
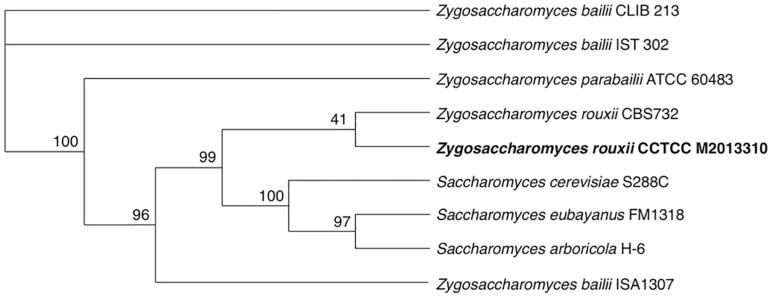
A phylogenetic tree for strain M2013310, as constructed using Orthofinder based on single-copy orthologs.

The phylogenetic position of M2013310 was evaluated by analyzing eight reference genome sequences from related yeast strains. The phylogenetic tree shows that all strains of the *Saccharomyces* genus and *Zygosaccharomyces rouxii* formed a very tight cluster adjacent to other *Zygosaccharomyces* species. The M2013310 strain formed a branch with *Z. rouxii* CBS732 and both species showed separation from the clade that was phylogenetically linked to *Saccharomyces* species (Figure 2). These results showed that M2013310 is more closely related to *Z. rouxii* species, which had also been proved by phylogenetic tree based on 26S rDNA (Supplementary Figure S1).

### Determination of Volatile Flavor Components

**FIGURE 3:**
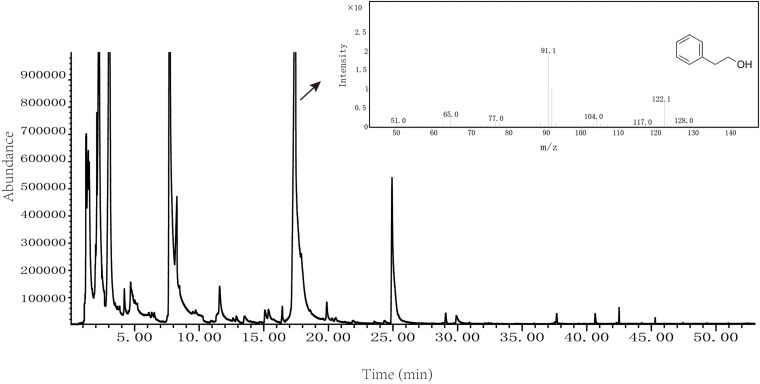
Volatile flavor compounds produced by *Z. rouxii* M2013310.

Volatile flavor compounds produced by *Z. rouxii* M2013310 were analyzed by solid phase micro extraction-mass spectrometry; the resultant spectrum is show in Figure 3. We also used spectrometry to identify total ion chromatograms for the volatile flavor components of *Z. rouxii* M2013310, including 16 types of alcohols, 2 types of phenols, 9 types of esters, 6 types of aldehydes, 8 types of ketones, 11 types of acids, 8 types of heterocyclic compounds, and 4 types of alkanes. Some important alcohol compounds (acetic acid, 2-phenylethyl ester, 3-methyl-1-butanol, and 2-phenylethanol) were detected in 7.2%, 11%, 13.2%, and 24.3%, of the total number of volatile flavor compounds, respectively (Supplementary Table S4). These results indicated that *Z. rouxii* M2013310 was capable of producing 2-PE.

**FIGURE 4:**
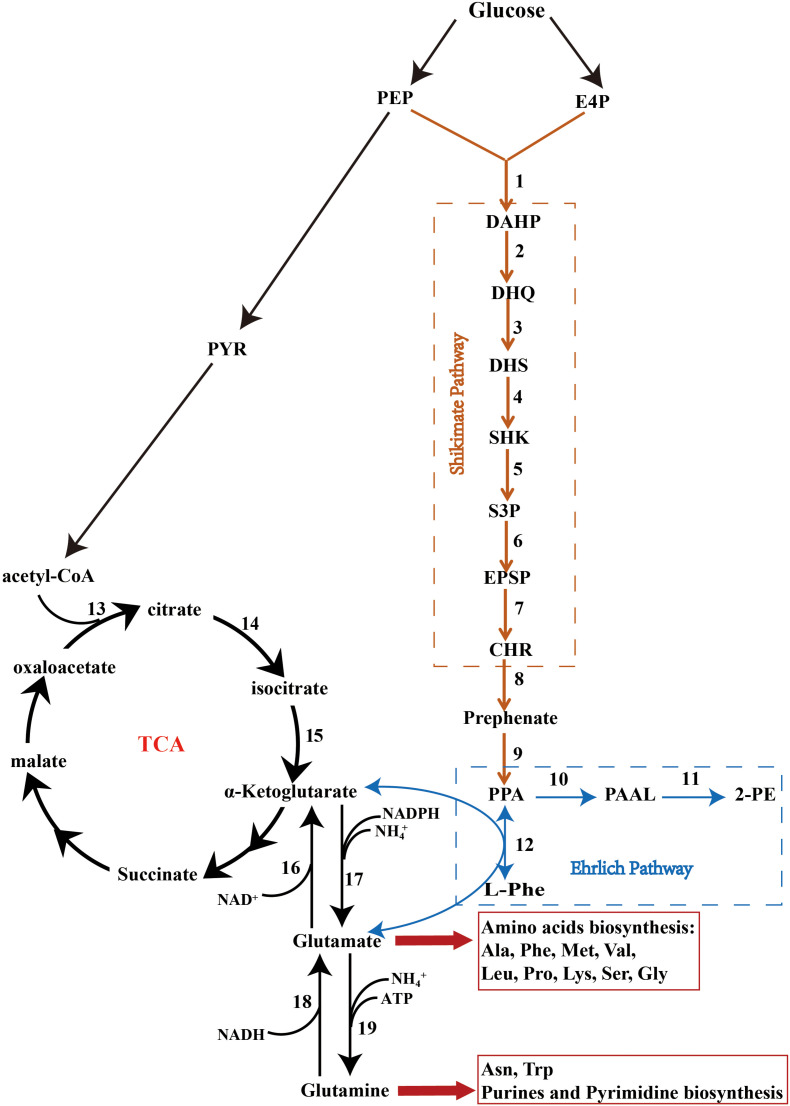
The Shikimate, Ehrlich, and cinnamate pathways. PEP, phosphoenolpyruvate; PYR, pyruvate; E4P, erythrose-4-phosphate; DAHP, 3-deoxy-D-arabinoheptulosonate; DHQ, 3-dehydroquinate; DHS, 3-dehydroshikimate; SHK, shikimate; S3P, shikimate-3-phosphate; EPSP, 5-enolpyruvyshikimate-3-phosphate; CHR, chorismate; PPA, phenylpyruvate; PAAL, phenylacetaldehyde; 2-PE, 2-phenylethanol; L-Phe, L-phenylalanine; Ala, alanine; Phe, phenylalanine; Met, methionine; Val, valine; Leu, leucine; Pro, proline; Lys, lysine; Ser, serine; Gly, glycine; Asn, asparagine; Trp, tryptophan. The factors marked 1–19 are listed in Supplementary Table S3.

### Analysis of the Pathway Used to Synthesize 2-Phenylethanol

KEGG pathway analysis showed that *Z. rouxii* M2013310 harbors the glycolysis, tricarboxylic acid (TCA), Shikimate, and Ehrlich pathways (Figure 4 and Supplementary Table S3) (Kanehisa et al., 2013). In the presence of preferred nitrogen sources, 2-PE was produced by *de novo* synthesis via the Shikimate pathway. Phosphoenolpyruvate (PEP), and erythrose-4-phosphate (E4P), arising from the glycolysis and pentose-phosphate pathways, respectively, are catalyzed to synthesize 1 by *AROF*, *AROG* and *AROH*, which encode 3-deoxy-7-phosphoheptulonate synthase. Phenylpyruvate is then synthesized via a series of reactions and finally converted into 2-PE. The major limitation of the Shikimate pathway is that the glycolysis and pentose-phosphate pathways are mainly directed into the TCA cycle for cell growth rather than for the synthesis of 2-PE. In comparison, the yield of 2-PE is significantly improved when 2-PE is bio-transformed from L-Phe *via* the Ehrlich pathway. The *ARO9* gene encodes aromatic aminotransferases II while the *ARO8* gene encodes aromatic aminotransferases I; these enzymes catalyze the conversion of L-Phe to phenylpyruvate. However, the by-product of this process, glutamate, is produced during the transamination reaction. *GUDB*, *ROCG*, *GDH2* and *GDHA* encode glutamate dehydrogenase, and enzyme that catalyzes glutamate to synthesize a-ketoglutarate that is directed into the TCA, thus repressing the synthesis of 2-PE *via* the Ehrlich pathway. The *ARO10* gene encodes phenylpyruvate decarboxylase, a rate-limiting enzyme, which catalyzes the decarboxylation of phenylpyruvate to phenylacetaldehyde. *ADH* encode alcohol dehydrogenases that catalyze the reduction of phenylacetaldehyde to form 2-PE (Hazelwood et al., 2008).

The production of 2-PE is highly dependent on the source of nitrogen. Different sources of nitrogen can influence the expression of crucial genes by nitrogen catabolite repression (NCR) (Cooper, 2002). The uptake of non-preferred nitrogen will result in NCR; this will significantly diminish in the presence of preferred nitrogen sources, thus affecting the expression of the general amino acid permease GAP1p that is used to transport aromatic amino acid L-Phe into yeast cells (Sáenz et al., 2014; Wang Z. et al., 2017). *ARO8*, *ARO9*, and *ARO10*, are the predominant research targets for the Ehrlich pathway. *ARO8* is responsible for the biosynthesis of phenylalanine and tyrosine (Iraqui et al., 1998). The expression of *ARO9* is induced by aromatic amino acids; while *ARO9* and *ARO10* are NCR-sensitive genes; their expression levels are regulated by GATA factors consisting of Gln 3 and Gat 1 (Broach, 2012; Lee and Hahn, 2013). Therefore, when using L-Phe as a sole source of nitrogen, yeasts such as *S. cerevisiae* can achieve maximized yields of 2-PE. The identification of the 2-PE biosynthesis pathway, and the roles of specific genes in this pathway, will play an important role in improving the production of 2-PE in a range of commercial sectors.

### The Effect of Exogenous 2-PE on the Growth of *Z. rouxii* M2013310

**FIGURE 5:**
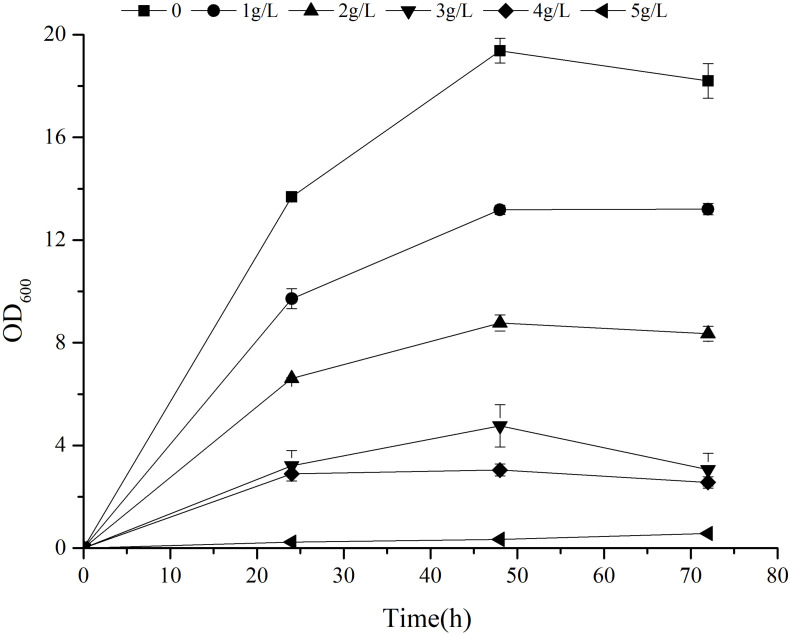
The effect of exogenous 2-PE on the growth of *Z. rouxii* M2013310.

Previous research has shown that 2-PE can enhance reactive oxygen species (ROS) accumulation, lipid peroxidation, and cell membrane damage, thus significantly inhibiting the production of 2-PE (Wang et al., 2020). The 2-PE yield of strain can be improved by the application of *in situ* product removal (ISPR) to alleviate the toxicity of 2-PE (Mierzejewska et al., 2017; Chreptowicz et al., 2018; Hua et al., 2010). Furthermore, the tolerance of strain to 2-PE plays an important role in alleviating product inhibition. To study the tolerance of *Z. rouxii* M2013310 with regards to 2-PE, we cultivated this yeast strain in YEPD supplemented with exogenous 2-PE to final concentrations of 1 g/L, 2 g/L, 3 g/L, 4 g/L and 5 g/L. After 72 h of fermentation, the cell density in the control group control was 7.11- and 32- fold higher than the density of cells in YEPD containing 4 g/L and 5 g/L 2-PE. At concentration of 5 g/L, the growth of the strain was significantly inhibited (Figure 5). These results showed that *Z. rouxii* M2013310 is tolerant to 2-PE up to a concentration of 4 g/L.

### The Effect of Different Nitrogen Sources on Growth and 2-PE Biosynthesis in *Z. rouxii* M2013310

To further investigate the pathway used to synthesize 2-PE, we inoculated *Z. rouxii* M2013310 into three different culture media: M3 (Phe), M3 (NH_4_^+^), and M3 (Phe + NH_4_^+^). The M3 (Phe) and M3 (NH_4_^+^) media use L-Phe and ammonium sulfate as the sole source of nitrogen, respectively. When cultured in M3 (Phe) media, *Z. rouxii* M2013310 used Ehrlich pathway to bio-transform L-Phe into 2-PE. When grown in M3 (NH_4_^+^) media, the yeast produced 2-PE by *de novo* synthesis (Etschmann et al., 2002, 2004). When grown in M3 (Phe) and M3 (NH_4_^+^) media, *Z. rouxii* M2013310 entered the stationary phase at 36 h; OD_600_ reached 13.4 and 11.25, respectively. However, the production of 2-PE in M3 (Phe) was 942-fold higher than that in M3 (NH_4_^+^). The highest yield of 2-PE, without the application of in suit product removal (ISPR), was 3.58 g/L in M3 (Phe) medium. This strain exhibited a four-fold higher capacity to produce 2-PE than *Z. rouxii* CBS 5717 (Etschmann et al., 2003). Collectively, these data indicate that the biotransformation of L-Phe to 2-PE is a key process in the production of 2-PE.

**FIGURE 6:**
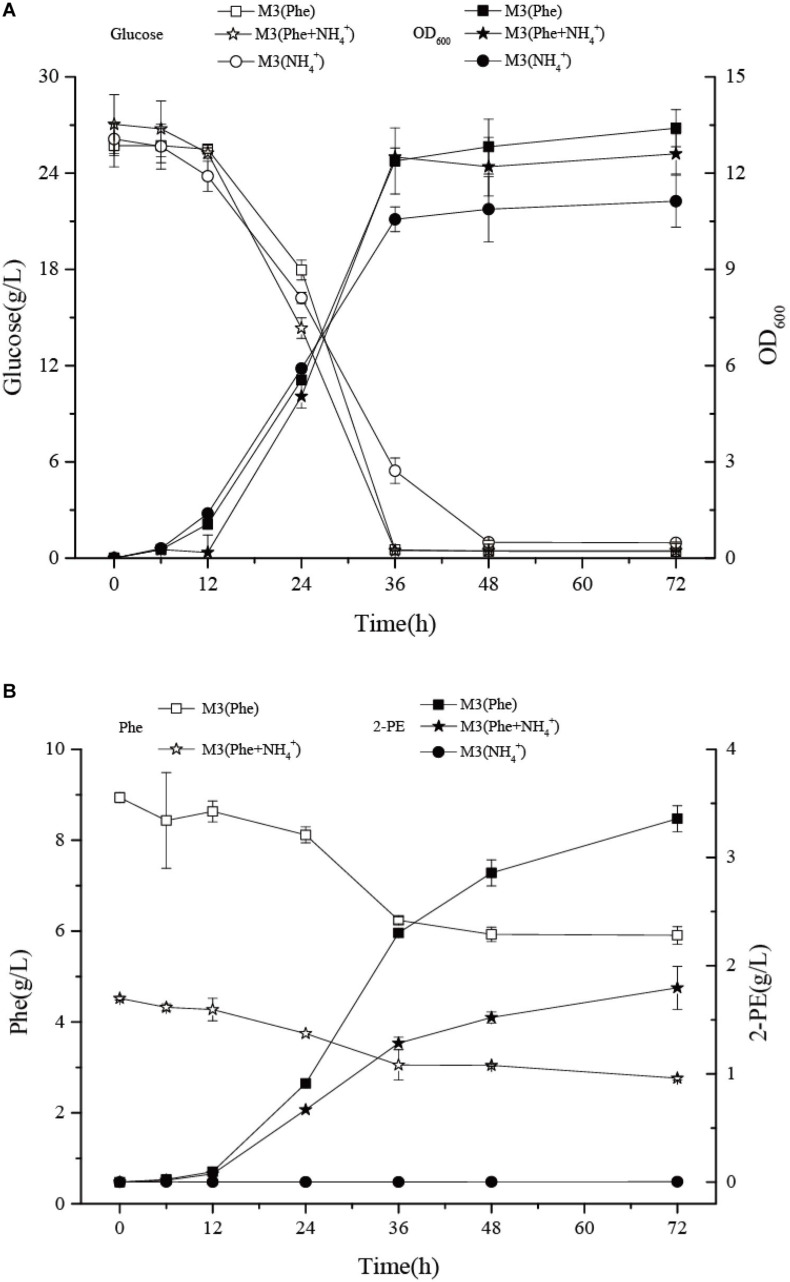
The effects of different nitrogen sources on the growth **(A)** and 2-PE synthesis of *Z. rouxii* M2013310 **(B)**.

Data relating to cell density and glucose consumption for the yeast were similar when cultivated in either M3 (Phe) or M3 (Phe + NH_4_^+^) medium, thus indicating that the co-existence of ammonium sulfate and L-Phe did not affect the growth of *Z. rouxii* M2013310. The concentration of 2-PE in M3 (Phe) was two-fold higher than that in M3 (Phe + NH_4_^+^) after 72 h of fermentation; we anticipated that the production of 2-PE would have continued to increase after this timepoint. During the adaptive period and the log phase, the strains grown in M3 (Phe) and M3 (Phe + NH_4_^+^) synthesized 2.3 g/L and 1.28 g/L of 2-PE, respectively. The 2-PE synthesis ability in M3 (Phe) and M3 (Phe + NH_4_^+^) was 64 mg/L/h and 36 mg/L/h, respectively. The consumption of L-Phe was 2.67 g/L and 1.45 g/L, respectively, with a consumption rate of 74 mg/L/h and 40 mg/L/h. After 36 h of fermentation, yeast cells entered the stationary phase and the consumption of L-Phe decreased notably. The consumption of L-Phe was 0.33 g/L and 0.29 g/L after 36 h and 72 h of culture, respectively, with a consumption rate of 9.2 mg/L/h and 8.1 mg/L/h (Figures 6A,B). The concentration of 2-PE in M3 (Phe) and M3 (Phe + NH_4_^+^) increased, to 1.28 g/L and 0.51 g/L, respectively, producing 35.5 mg/L/h and 14.2 mg/L/h of 2-PE, respectively. In M3 (Phe) and M3 (Phe + NH_4_^+^) media, the yield of 2-PE was 5.3- and 2.3-fold higher than the maximum theoretical concentration of the product that could be achieved by the bioconversion of the remaining L-Phe after 36 h of fermentation. This indicated that there may be additional enzymes that are activated to promote the biotransformation of intermediates in the Ehrlich pathway to synthesize 2-PE, or that the strain also synthesized 2-PE via the Shikimate pathway. These remain for future studies to determining the concentration of phenylpyruvate and phenylacetaldehyde and the activity of related enzyme (Wang P. et al., 2017; Wang Z. et al., 2017). The transformation ratio of the non-genetically modified strain of *Z. rouxii* M2013310 was 0.53 mol/mol. Furthermore, a strain of *S. cerevisiae* S288c (0.5 mol/mol) that over-expresses *ARO8* and *ARO10* (Yin et al., 2015), exhibited a clear advantage with regards to 2-PE production.

### The Relative Expression Levels of *GAP1*, *ARO8*, *ARO9*, and *ARO10*, in *Z. rouxii* M2013310 When Cultured in Different Media

qRT-PCR analysis showed that the mRNA levels of general amino acid permease (*GAP1*) in M3 (Phe) and M3 (NH_4_^+^) were 124- and 86-fold higher than that in M3 (Phe + NH_4_^+^) after 24 h of fermentation. When L-Phe and ammonium sulfate were used as nitrogen sources, GAP1p activity fell rapidly although L-Phe uptake and bioconversion continued (Figure 6B). These results suggested that GAP1p was not the only permease involved in L-Phe uptake (Sáenz et al., 2014).

**FIGURE 7:**
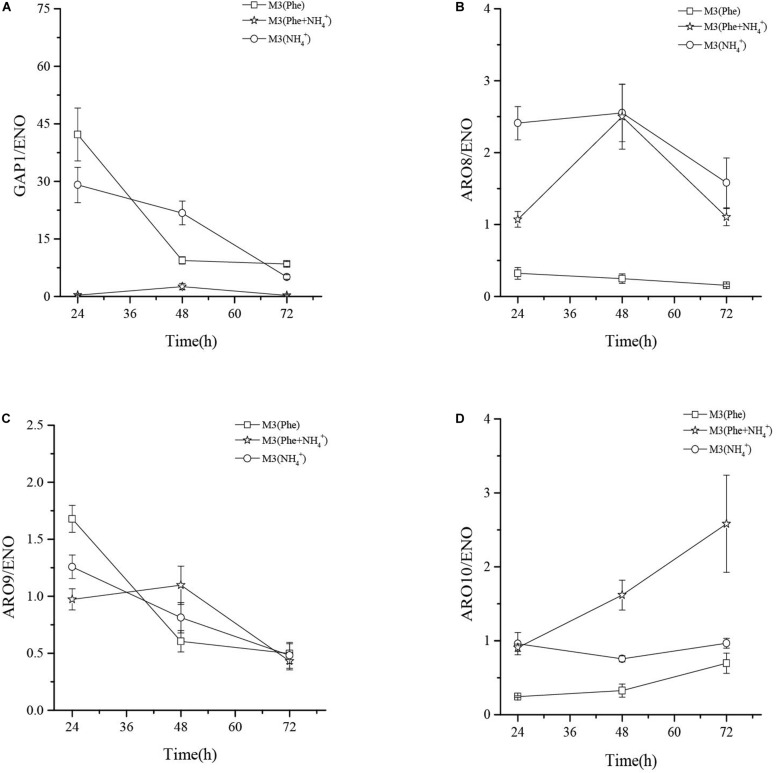
The mRNA relative expression levels of *GAP1* **(A)**, *ARO8* **(B)**, *ARO9* **(C)**, and *ARO10* **(D)** of *Z. rouxii* M2013310 in three different culture media.

The mRNA levels of *ARO9* were similar when compared in three different culture media, thus indicating that the concentration of ammonium ions in the media had little effect on the expression of *ARO9*. The expression levels of *ARO8* mRNA in M3 (NH_4_^+^) and M3 (Phe + NH_4_^+^) were similar after 48 h of fermentation and the mRNA levels of *ARO8* in the two types of culture media were 10-fold higher than that in M3 (Phe). The mRNA levels of *ARO10* (2-keto acid decarboxylase) in M3 (NH_4_^+^) were significantly higher than those in M3 (Phe + NH_4_^+^) and M3 (Phe) media. The expression levels of *ARO8* and *ARO10* mRNA in M3 (Phe) were lower than in the other two media. However, when comparing the concentration of 2-PE after fermentation of the strain in three different media, we found that the highest concentration of 2-PE was produced by the strain grown in M3 (Phe) media (Figure 7). This data suggests that this strain of yeast may bio-transform L-Phe to 2-PE via an alternative pathway, or these crucial enzymes (ARO8p, ARO10p, and GAP1p) may be regulated by other genes such as *AGP1, BAP2*, and *PDC* (Kim et al., 2014; Sáenz et al., 2014). However, this hypothesis needs to be verified by future research studies.

## Conclusion

In the present study, we used PacBio sequencing technology to characterize the biological properties and genomic features of *Z. rouxii* M2013310, a strain of yeast, that we isolated from chili sauce. In addition, we found that *Z. rouxii* M2013310 was capable of synthesizing 2-PE in YEPD medium. We used three different types of culture media to investigate the pathway by which *Z. rouxii* M2013310 synthesizes 2-PE. The highest concentration of 2-PE synthesized by *Z. rouxii* M2013310 was 3.58 g/L in the M3 (Phe) medium. Transamination and decarboxylation are essential for 2-PE synthesis via the Ehrlich pathway. Similarly, *ARO8*, *ARO9*, and *ARO10*, genes are all crucial L-Phe biotransformation. The mRNA levels of *ARO8* and *ARO10* in *Z. rouxii* M2013310 grown in M3 (Phe) were lower than when the same yeast strain was grown in M3 (NH_4_^+^) or M3 (Phe + NH_4_^+^) media. Our data suggest that the Ehrlich pathway may not be the only pathway involved in the synthesis of 2-PE in M3 (Phe) medium of *Z. rouxii* M2013310, although this requires further verification. We identified a promising target strain (*Z. rouxii* M2013310) that can be used to improve the commercial production of 2-PE, which is firstly proposed.

## Accession Numbers

*Zygosaccharomyces rouxii* M2013310 has been deposited in China Center for Type Culture Collection under the number: CCTCC M2013310. The assembly and raw sequencing data have been deposited in GenBank under BioProject accession PRJNA577023, WHVI01000000 for genome assembly data, and SRR10260307 for genomic PacBio sequencing data.

## Data Availability Statement

The datasets presented in this study can be found in online repositories. The names of the repository/repositories and accession number(s) can be found in the article/Supplementary Material.

## Author Contributions

JD and XC conceived the research. KL, NS, WY, HX, QY, XZ, XL, ZW, LY, and SY conducted all experiments. JD and KL wrote the manuscript. All authors edited and approved the manuscript.

## Conflict of Interest

The authors declare that the research was conducted in the absence of any commercial or financial relationships that could be construed as a potential conflict of interest.
