# Supplementary material for: Zygosaccharomyces rouxii, an Aromatic Yeast Isolated From Chili Sauce, Is Able to Biosynthesize 2-Phenylethanol via the Shikimate or Ehrlich Pathways
Source: Front Microbiol. 2020 Oct 29;11:597454. doi: 10.3389/fmicb.2020.597454 (PMC7673420; doi:10.3389/fmicb.2020.597454)
Supplement: Supplementary file 1 [file Data_Sheet_1.PDF]

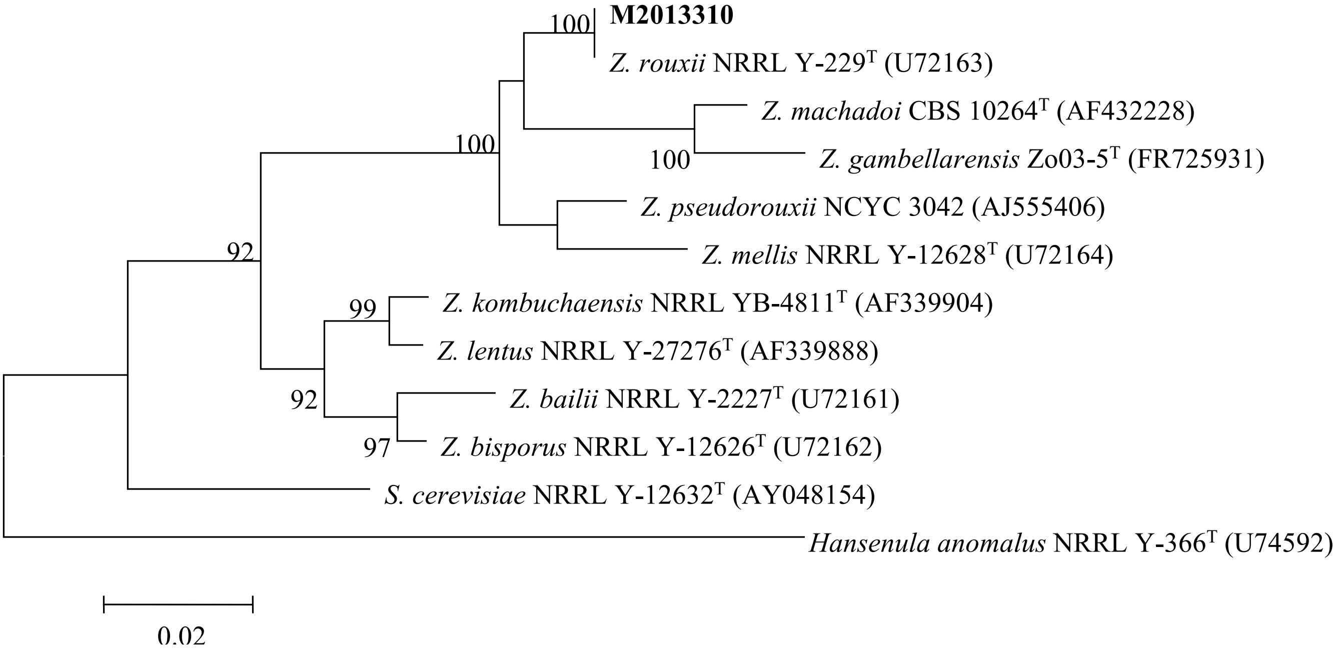

Supplementary Figure S1

Neighbour-joining phylogenetic tree, based on 26S rRNA gene sequences, showing the relationships of strain M2013310 and representative members of the phylum *Fungi*. Bootstrap values (1000 replications) are shown as percentages at each node only if they are 70% or greater. Bar, 0.02 substitutions per nucleotide position.

Method

Phylogenetic analysis based on 26S rRNA gene was performed by using the software package MEGA version 5.0 (Tamura et al. 2011) after multiple alignment of the data via CLUSTAL_X (Thompson et al. 1997). Phylogenetic trees were constructed by neighbor-joining (Saitou and Nei 1987). Evolutionary distances for the neighbor-joining algorithm were calculated with Kimura’s two-parameter method (Kimura 1980) and the topology of the neighbor-joining tree was evaluated by bootstrap analysis on the basis of 1000 replications (Felsenstein 1985). Close-neighbor-interchange (search level = 2, random additions = 100) was applied in the maximum-parsimony analysis. Sequences from *Saccharomyces cerevisiae* (AY048154) and *Pichia anomalus* (U74592) were included as the outgroup.

References

Tamura K, Peterson D, Peterson N, Stecher G, Nei M, Kumar S (2011) MEGA5: molecular evolutionary genetics analysis using maximum likelihood, evolutionary distance, and maximum parsimony methods. *Molecular biology and evolution* 28 (10):2731-2739. doi:10.1093/molbev/msr121

Thompson JD, Gibson TJ, Plewniak F, Jeanmougin F, Higgins DG (1997) The CLUSTAL_X windows interface: flexible strategies for multiple sequence alignment

aided by quality analysis tools. *Nucleic acids research* 25 (24):4876-4882
Saitou N, Nei M (1987) The neighbor-joining method: a new method for reconstructing phylogenetic trees. *Molecular biology and evolution* 4 (4):406-425
Kimura M (1980) A simple method for estimating evolutionary rates of base substitutions through comparative studies of nucleotide sequences. *Journal of molecular evolution* 16 (2):111-120
Felsenstein J (1985) Confidence Limits on Phylogenies: An Approach Using the Bootstrap. *Evolution* 39 (4):783-791. doi:10.2307/2408678
