# Supplementary material for: Zygosaccharomyces rouxii, an Aromatic Yeast Isolated From Chili Sauce, Is Able to Biosynthesize 2-Phenylethanol via the Shikimate or Ehrlich Pathways
Source: Front Microbiol. 2020 Oct 29;11:597454. doi: 10.3389/fmicb.2020.597454 (PMC7673420; doi:10.3389/fmicb.2020.597454)
Supplement: Supplementary file 4 [file Table_3.DOC]

Supplementary Table S3.The genes of Shikimate, Ehrlich, and cinnamate pathways for strain M2013310.

| Number | KO ID | EC ID | KEGG Gene Name | Definition | Gene Count | M2013013  Genome ID |
| --- | --- | --- | --- | --- | --- | --- |
| 1 | K01626 | 2.5.1.54 | aroF, aroG, aroH | 3-deoxy-7-phosphoheptulonate synthase | 4 | g1574.t1_1  g1706.t1_1  g4372.t1_1  g8308.t1_1 |
| 2, 3, 4, 5, 6 | K13830 | 4.2.3.4 4.2.1.10 1.1.1.25 2.7.1.71 2.5.1.19 | Aro1 | Pentafunctional AROM polypeptide | 1 | g7603.t1_1 |
| 7 | K01736 | 4.2.3.5 | Aro2 | chorismate synthase | 2 | g4225.t1_1  g8589.t1_1 |
| 8 | K01850 | 5.4.99.5 | Aro7 | chorismate mutase | 2 | g2982.t1_1  g6749.t1_1 |
| 9 | K04518 | 4.2.1.51 | pheA2 | prephenate dehydratase | 2 | g2445.t1_1  g6209.t1_1 |
| 10 | K12732 | 4.1.1.- | ARO10 | phenylpyruvate decarboxylase | 2 | g5160.t1_1  g843.t1_1 |
| 11 | K13951 | 1.1.1.1 | ADH1_7 | alcohol dehydrogenase 1/7 | 16 | g3633.t1_1  g3999.t1_1  g543.t1_1  g2653.t1_1  g2842.t1_1  g3529.t1_1  g3543.t1_1  g442.t1_1  g456.t1_1  g5121.t1_1  g5123.t1_1  g6414.t1_1  g6603.t1_1  g7172.t1_1  g805.t1_1  g807.t1_1 |
|  | K00002 | 1.1.1.2 | AKR1A1, adh | alcohol dehydrogenase (NADP+) | 2 | g1088.t1_1  g5412.t1_1 |
| 12 | K14454 | 2.6.1.1 | GOT1 | aspartate aminotransferase, cytoplasmic | 5 | g5702.t1_1  g304.t1_1  g3387.t1_1  g5116.t1_1  g800.t1_1 |
|  | K00815 | 2.6.1.5 | TAT | tyrosine aminotransferase | 2 | g2569.t1_1  g6334.t1_1 |
|  | K00817 | 2.6.1.9 | hisC | histidinol-phosphate aminotransferase | 2 | g4265.t1_1  g5645.t1_1 |
|  | K00832 | 2.6.1.57 | tyrB | aromatic-amino-acid transaminase | 2 | g2569.t1_1  g6334.t1_1 |
|  | K05821 | 2.6.1.58 2.6.1.28 | ARO9 | aromatic amino acid aminotransferase II | 2 | g5208.t1_1  g882.t1_1 |
| 13 | K01647 | 2.3.3.1 | CS, gltA | citrate synthase | 7 | g1840.t1_1  g4273.t1_1  g4506.t1_1  g5652.t1_1  g5653.t1_1  g5859.t1_1  g8039.t1_1 |
| 14 | K01681 | 4.2.1.3 | ACO, acnA | aconitate hydratase | 2 | g1578.t1_1  g8305.t1_1 |
| 15 | K00031 | 1.1.1.42 | IDH1, IDH2, icd | isocitrate dehydrogenase | 4 | g176.t1_1  g3260.t1_1  g5887.t1_1  g8067.t1_1 |
|  | K00030 | 1.1.1.41 | IDH3 | isocitrate dehydrogenase (NAD+) | 2 | g7407.t1_1  g7774.t1_1 |
| 16 | K00260 | 1.4.1.2 | gudB, rocG  GDH2 | glutamate dehydrogenase | 2 | g2722.t1_1  g6485.t1_1 |
|  | K00262 | 1.4.1.4 | gdhA | glutamate dehydrogenase (NADP+) | 2 | g2336.t1_1  g6100.t1_1 |
| 17 | K00265 | 1.4.1.13 | gltB  gltD | glutamate synthase (NADPH) large chain | 2 | g3666.t1_1  g576.t1_1 |
| 18 | K00264 | 1.4.1.14 | GLT1 | glutamate synthase (NADH) | 2 | g3666.t1_1  g576.t1_1 |
| 19 | K01915 | 6.3.1.2 | glnA, GLUL | glutamine synthetase | 2 | g1373.t1_1  g8505.t1_1 |
