# Supplementary material for: Zygosaccharomyces rouxii, an Aromatic Yeast Isolated From Chili Sauce, Is Able to Biosynthesize 2-Phenylethanol via the Shikimate or Ehrlich Pathways
Source: Front Microbiol. 2020 Oct 29;11:597454. doi: 10.3389/fmicb.2020.597454 (PMC7673420; doi:10.3389/fmicb.2020.597454)
Supplement: Supplementary file 5 [file Table_4.DOCX]

Supplementary Table S4: Major volatile flavor compounds of M2013310.

| **Retention Time(min)** | **Name** | **CAS number** |
| --- | --- | --- |
| 1.416 | Ethanol | 000064-17-5 |
| 2.274 | 1-Butanol | 000071-36-3 |
| 3.047 | 3-Methyl-1-butanol | 000123-51-3 |
| 7.745 | 1-Pentyl acetate | 000628-63-7 |
| 17.460 | 2-Phenylethanol | 000060-12-8 |
| 24.927 | 2-phenylethyl ester | 000103-45-7 |
